# Walking Green: Developing an Evidence Base for Nature Prescriptions

**DOI:** 10.3390/ijerph16224338

**Published:** 2019-11-07

**Authors:** Elizabeth P.D. Koselka, Lucy C. Weidner, Arseniy Minasov, Marc G. Berman, William R. Leonard, Marianne V. Santoso, Junia N. de Brito, Zachary C. Pope, Mark A. Pereira, Teresa H. Horton

**Affiliations:** 1 Department of Anthropology, Northwestern University, 1810 Hinman Avenue, Evanston, IL 60208, USALucyWeidner2021@u.northwestern.edu (L.C.W.); arseniy.minasov@northwestern.edu (A.M.); w-leonard1@northwestern.edu (W.R.L.); 2 Department of Psychology, University of Chicago, 5848 S. University Avenue, Chicago, IL 60637, USA; bermanm@uchicago.edu; 3 Division of Nutritional Sciences, Cornell University, 244 Garden Ave, Ithaca, NY 14853, USA; mvs@northwestern.edu; 4 School of Public Health, Division of Epidemiology and Community Health, 1300 S. 2nd Street, Room 300 West Bank Office Building, Minneapolis, MN 55454, USA; nogue013@umn.edu (J.N.d.B.); popex157@umn.edu (Z.C.P.); perei004@umn.edu (M.A.P.)

**Keywords:** green exercise, nature prescriptions, nature Rx, physical activity, anxiety, directed-attention

## Abstract

Although the health benefits of exercise and exposure to nature are well established, most evidence of their interaction comes from acute observations of single sessions of activity. However, documenting improved health outcomes requires ongoing interventions, measurement of multiple outcomes, and longitudinal analyses. We conducted a pilot study to guide the development of a protocol for future longitudinal studies that would assess multiple physiological and psychological outcomes. Herein, we report psychological outcomes measured from thirty-eight participants before and after three conditions: a 50 min walk on a forest path, a 50 min walk along a busy road, and a period of activities of daily living. Changes in positive and negative affect, anxiety, perceived stress, and working memory are reported. We benchmark these results to existing studies that used similar protocols and also identify elements of the protocol that might impair recruitment or retention of subjects in longer-term studies. Linear mixed-models regression revealed that walking improved psychological state when compared to activities of daily living, regardless of walk environment (*p* < 0.05). Comparison of mean differences showed that forest walks yielded the largest and most consistent improvements in psychological state. Thus, despite a protocol that required a 3.5 h time commitment per laboratory visit, the beneficial effects of walking and exposure to a forested environment were observed.

## 1. Introduction

Mental health diagnoses and the need for mental health treatments have increased substantially in recent decades. Current estimates suggest that 20% of adults experience a mental illness in a given year, and 4.6% experience a serious mental illness in 2018 [1]. Some of the increase in diagnoses of mental illness across socio-economic groups has been attributed to the stresses of urbanization and lack of contact with nature [2,3,4,5]. In response, awareness of the relationship between mental health and the structure of the built environment has grown worldwide, leading to a corresponding increase in research on the social and health benefits of urban greenspace. 

Urban greenspace is defined as areas with predominant vegetation (e.g., trees, grasslands, or water features), whether naturally occurring or man-made spaces [6,7]. The American Public Health Association has recommended that public health practitioners collaborate with urban planners, parks and recreation departments, and others to increase access to greenspace and encourage people to be more active outdoors [8,9]. Similarly, the United Nations made the unprecedented move in 2015 to include promotion of mental health in their Sustainable Development Agenda and proposed that access to greenspace in urban areas be incorporated into public health initiatives [10,11]. A grass roots movement has developed to encourage physicians and other health care providers to encourage patients to engage in physical activity in greenspaces by writing “nature”, “park”, or “green” prescriptions (hereafter “Nature Rx”) [12,13,14,15,16]. Nature Rx is an example of a non-pharmacological or social prescription intended to increase physical activity and increase time outside [16]. However, evidence documenting the specific health conditions that would benefit from the appropriate “dose” of nature and how different populations may respond to Nature Rx is lacking [17,18,19,20].

Several mechanisms have been proposed to yield improvements in psychological and physiological outcomes in response to exposure to greenspace independently of increased physical activity. These diverse mechanisms include, but are not limited to: psychological stress reduction; attention restoration; exposure to cleaner air and secondary plant compounds (phytoncides); and improved social networks [9,19,20,21,22,23,24,25,26,27,28,29,30,31]. Population-based cross-sectional studies show that living in or near urban greenspaces is associated with a lower incidence of psychiatric morbidity (including anxiety, and both self-reported and objective stress measures), as well as cardiometabolic disease, after controlling for measures of socioeconomic status, demographic factors, and physical activity [32,33,34,35,36]. A limited number of longitudinal studies also support the hypothesis that exposure to greenspace may promote improved health outcomes, such as self-reported mental health and the incidence of type 2 diabetes, independently of increased physical activity [37,38,39]. Thus, while numerous studies have long indicated that light-to-moderate physical activity has a strong positive association with health and well-being [40,41,42,43], greenspace exposure has unique characteristics that may confer added health benefits via multiple pathways for those completing physical activity in a green environment. 

Additional evidence supporting the hypothesis that exposure to greenspace adds to or synergizes with physical activity comes from experimental studies that measure a battery of mental and cognitive outcomes before and after physical activity in green and non-green environments [12,44,45]. Berman and colleagues demonstrated that, following a mentally fatiguing task, a 50–55 min walk in the forest, but not along city sidewalk, conferred improvements in attention and affect [46]. Bratman et al. similarly found that a 90 min walk in a forest led to reductions in rumination (i.e., the process of continuously thinking the same thoughts repeatedly, which is generally associated with anxiety and depression) and decreases in neural activity in brain regions associated with rumination [47]. Meanwhile, Park and colleagues found improvements in psychological and physiological (e.g., cortisol and heart rate variation) measures of stress when participants walked in, or simply sat and viewed a forest for 20 min, but not city streets [48,49]. 

Taken together, the preceding papers and others in the literature indicate that a single bout of exercise in greenspace results in greater improvements in acute measures of cognitive function, mood, and mental well-being, compared to similar amounts of exercise completed indoors or in urban areas [50,51,52]. In contrast, studies of walking interventions aimed at improving health outcomes typically last weeks or months [53,54,55,56,57,58]. Although studies comparing the effects of walks in green- versus non- greenspaces show dramatic changes in acute measures of psychological state, a research gap exists in our understanding of whether regular bouts of exercise in greenspace lead to greater improvement in long-term health outcomes and quality of life than the same amount of activity in built spaces.

The Walking Green Project (WG) was designed to engage and enrich the growing field of inquiry surrounding the Nature Rx movement. The project consists of two pilot studies one conducted at Northwestern University (NU) (ClinicalTrials.gov registration #NCT03950661) and one at the University of Minnesota (UMN) (e.g., de Brito et al., [59] ClinicalTrials.gov #NCT03442998). Both pilot studies were designed to inform the development of protocols for future studies of longer duration in populations with documented mental or physical health concerns. Of specific concern for the development of future protocols was that the burden imposed by taking multiple physiological and psychological measures at each visit might offset benefits of the proposed interventions. Therefore, the present paper examines a subset of data from WG-NU to provide a direct comparison to the study by Berman and colleagues [46]. Both WG-NU and Berman et al. [46] used similar study cohorts (convenience samples of healthy, young adults), similar exposures (50 min walks along a forest path and a busy road), and similar assessments (Positive and Negative Affect Scale, backward digit span test) [46]. WG-NU additionally assessed anxiety and perceived stress and collected data from sessions during which participants engaged in their normal daily activities (Activities of Daily Living, ADL) instead of study-related walking. We report on lessons learned in our efforts to recruit and retain equal numbers of men and women for a protocol that required multiple visits to the laboratory, some lasting up to 3.5 hours. 

## 2. Materials and Methods 

### 2.1. Ethics Statement

**Figure 1:**
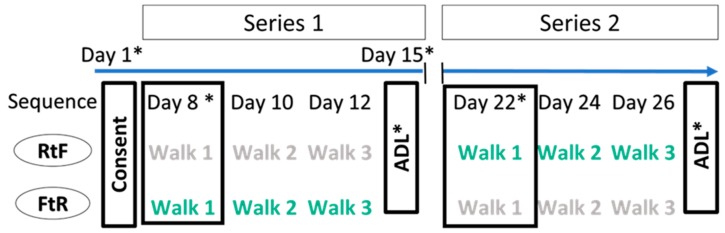
Timeline for the full Walking Green- Northwestern University (NU) study design. This report includes only those data collected on days 1, 8, 15, 22, and 29 as indicated by an asterisk (*). “Sequence” refers to the order in which walks were taken (Roadside to Forest (RtF) or Forest to Road (FtR). Grey text indicates roadside walks. Green text indicates forest walks.

This study was approved by the Northwestern University Institutional Review Board (approval number STU00201604) and was registered with ClinicalTrials.gov (NCT03950661). Written informed consent was obtained from each participant on their first visit to the laboratory, and before data collection (Figure 1, Day 1). Participants were compensated up to $207.00 US for their participation.

### 2.2. Participants

We recruited a convenience sample of undergraduate and graduate students and employees from Northwestern University and residents of Evanston, IL, USA and nearby communities using social media, email listservs, and paper flyers posted on campus and in the surrounding community. A specific recruitment goal was to enroll equal numbers of men and women in the study sample. To recruit a cohort comparable to previous studies [46,47,48] potential participants were screened against the following inclusion criteria: (1) between the ages of 18–35 years (mean + SD: 22.9+ 4.6), (2) fluent in English, and (3) healthy as assessed by their readiness to engage in moderate physical activity, which was assessed via the Physical Activity Readiness Questionnaire (PAR-Q+). Women who were pregnant or breastfeeding were excluded. 

Most participants were full-time students (graduate or undergraduate) at Northwestern University (Table 1). Reported household incomes ranged from under US $50,000 to over US $100,000 per year. Over 50% of the participants were Asian or Asian American (52.6%). Approximately 10.5% of the participants were Hispanic, 5.3% were African American, and 31.6% were non-Hispanic white. Only three participants spent their childhoods living in rural locations. At the time of the study, all participants lived and worked in urban or suburban areas around Evanston, IL, including northern Chicago, IL.

### 2.3. Study Design

Walking Green-NU used a within-subjects randomized crossover design (Figure 1). After providing informed consent, participants completed a brief survey to assess their psychological and cognitive states (hereafter, “survey”). Subsequently, each participant completed two series of three 50 min walks in a specified location, separated by a nine-day washout period between series (Figure 1). Each participant completed a series of three 50 min walks along a busy road and one series in a forest preserve, but the sequence (Roadside to Forest (RtF) or Forest to Road (FtR)) for walk locations was assigned randomly (Figure 1).

**Figure 2:**
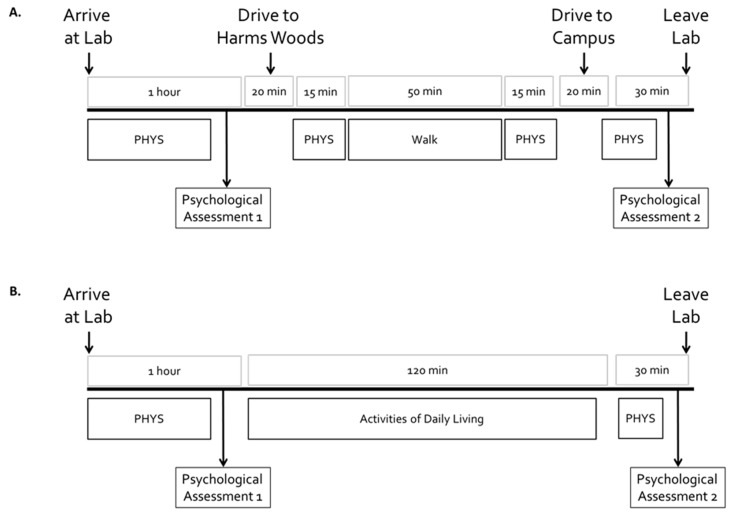
(**A**) Timeline for events on walking days (days 8 and 22) and (**B**) For Control days (days 15 and 29). PHYS indicates times when anthropometric and physiological assessments were conducted for which data will be reported separately. However, the occurrence of these additional assessments is included in this schematic to provide context about the activities and potential stress experiences by the participants.

This report includes only data collected on days 1, 8, 15, 22, and 29 of the study (Figure 1). The walks taken on days 8 and 22 are the first walks that each participant took in each location and thereby provided the most direct comparison to prior studies [46,47,48]. Prior to each walk participants came to the laboratory, where they completed the survey to assess their psychological and cognitive state and also engaged in measurement of physiological outcomes (Figure 2A). Participants were then driven to the start site for the walk; after the walk they were driven back to the laboratory where they repeated the survey in order to assess their post-walk psychological and cognitive state (Figure 2A). 

To provide control data about changes in psychological and cognitive state on days with no walks, participants came to the laboratory on days 15 and 29 and completed the survey but did not go for a study-related walk. Instead, participants left the laboratory to go about their activities of daily living (ADL) for 2 hours. Upon returning to the laboratory, participants repeated the survey (Figure 2B). 

To accommodate participants’ work and class schedules, some participants did their surveys and walks, or ADL sessions, starting in the morning (between 08:00 and 09:00 local time), others in the afternoon (between 13:00 and 14:00 local time). However, the time of day for each participant was held constant to control for diurnal variation in responses.

### 2.4. Location and Instructions for Walks

**Figure 3:**
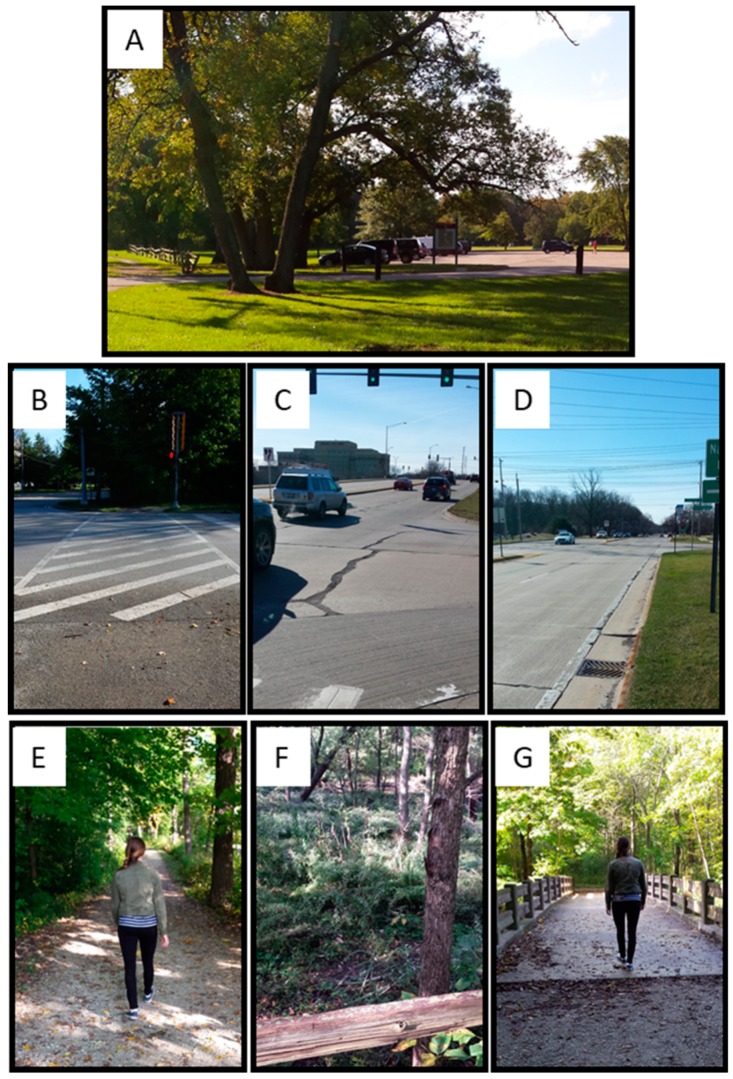
Starting location for all walks was a parking lot in the Forest Preserve. (**B**–**D**). Views along the Roadside walk. (**E**–**G**). Views along the Forest Path.

After completing the pre-walk survey, one to four participants were driven from the laboratory to the starting point for the walks; a drive of approximately 10 km (~20 min; one-way). The duration of the drive is consistent with the Forest Preserve District of Cook County’s goal to ensure that 75% of county residents can access forest preserves in 20 min by car or 45 min by public transportation [60]. Forest and roadside walks both began and ended in the parking lot of Harms Woods (George F. Nixon Woods, Figure 3A), one of the Forest Preserves of Cook County, IL, located at the intersection of Harms Road and Old Orchard Road in Glenview, IL. Participants assigned to walk along the roadside crossed Harms Road (Figure 3B) and walked east, on the sidewalk of Old Orchard Road (Figure 3C,D). This road is a busy 4 lane road that passes over Interstate 94 as it approaches a large shopping mall approximately 1 km away. Participants assigned to walk in the forest walked north from the parking lot on a well-groomed gravel trail into the woods along the North Branch Trail (Figure 3E), which follows the Chicago River (Figure 3F). After walking north for approximately 500 m, they crossed a footbridge over the river then walked south on the graveled trail for approximately 750 m (Figure 3G). Distances walked along both routes varied depending on each participants’ pace. 

Participants were instructed to walk the assigned route for 25 min and then return by the same route. All participants walked alone. If multiple participants had been driven to the walk site together, their start times were staggered so they were not walking in pairs or groups. Participants did not carry cell phones or other mobile devices during the walks, and they were provided a stopwatch to time their walks. Participants controlled their own walking pace, were discouraged from running, and were asked not to sit down at any point, but simply to walk continuously. After a nine-day washout period, participants walked in the other location.

### 2.5. Instructions for “Activities of Daily Living” Sessions

On days 15 and 29 of the study (Figure 1), participants came to the laboratory to provide data about their psychological wellness on a control, non-walking day (i.e., Activities of Daily Living, ADL). On these days, they arrived at the laboratory and completed the same survey as completed on walking days. After completing the survey, participants were instructed to go about their normal daily activities for two hours. The two-hour period approximated the amount of time required to drive to and from the walk site and take a walk. Participants were asked to refrain from vigorous physical activity, but otherwise could engage in their routine ADLs (e.g., studying, regular work, household chores, etc.). After two hours, participants returned to the laboratory and repeated the survey. 

Completion of all the assessments and interventions for the days reported in this paper required approximately 3.5 hours per day (Figure 2A,B). The total time commitment for each participant over the course of nine visits to the laboratory was approximately 23 hours (Figure 1). Because of the large time commitment required from the participants, we describe our experience with recruitment and retention in narrative form in Results. 

### 2.6. Outcomes of Interest

Before and after each walk or an ADL control session, participants completed a brief survey that contained psychological scales and a test of attention and working memory. These surveys are labelled as Psychological Assessments 1 and 2 in Figure 2 and are described below.

- The Positive and Negative Affect Schedule (PANAS) is a self-report questionnaire that consists of two 10-item scales that measure both positive and negative affect. Positive affect reflects the extent to which a person feels enthusiastic, active, and alert. Negative affect denotes subjective experiences of distress and unpleasurable engagement and subsumes a verity of unpleasant mood states, including anger, disgust, and fear [61,62].
- The State and Trait Anxiety Inventory (STAI) is a self-report measure that indicates the intensity of feelings of anxiety [63,64]. The STAI distinguishes between state anxiety—a temporary condition experienced in specific situations—and trait anxiety, which is a general tendency to perceive situations as threatening. For this analysis, we focused on STAI-state scores because they aligned with the short-term changes in anxiety we planned to assess.
- Cohen’s Perceived Stress Scale (PSS-10) is a measure of the degree to which situations in one’s life are appraised as stressful. Items were designed to examine how unpredictable, uncontrollable, and overloaded respondents find their lives. The scale also includes several direct queries about current levels of perceived stress [65,66].
- Visual Backward Digit Span Test (vBDS) is a component of the Wechsler Adult Intelligence Scale and is used to assess attention and working memory [67]. This test has been used to evaluate the role of exposure to greenspace in reducing mental fatigue as indicated by increased working memory capacity [46,68,69,70,71]. The test was administered by computer and was non-adaptive (i.e., sequences did not change in response to a participant’s responses). Participants viewed a series of digits at one-second intervals on a computer screen, then were asked to enter the sequence in reverse order using a computer keyboard. The test started with a sequence of three digits and increased to nine digits, with each series length repeated twice. Providing the correct response for each sequence was scored as one point, thus the maximum score possible was 14.

### 2.7. Statistical Analysis

A statistical power analysis was performed using GPower 3.1 [72,73] to estimate sample size for a comparison of differences between two dependent means (matched pairs). Because different outcome measures may yield different effect sizes, sample sizes were estimated for small (Cohen’s d = 0.25) and medium (Cohen’s d = 0.50) effect sizes assuming alpha = 0.05 and power = 0.80 [74]. Assumption of a small effect size yielded an estimated total sample size of 128, in contrast assumption of a medium effect size suggest a total sample size of 34. Based on data from Berman [46] which used a sample size of 38 and indicated a medium effect size for the effect of location on working memory (backward digit span), we selected a target sample size of 40. Similar or smaller sample sizes were used by Bratman et al. [47] (N = 19) and Park et al. [48] (N = 12). Forty-one participants provided informed-consent to participate in the study. Of these, 38 participants completed the full study protocol (93% completion rate): 20 women and 18 men. Because dropouts were due to reasons unrelated to the study (i.e., changes in course or work schedules, illness), these participants’ data were not included in these analyses (a per-protocol analysis) [75]. 

To verify that assignment of participants to the two sequences yielded equivalent groups, demographic variables, and psychological and working memory scores were compared by Sequence assignment. Demographic data were analyzed using Student’s t-test or the Chi-squared test as appropriate for the data (Table 1 and Table 2). Data presented in Table 1 and Table 2 demonstrated that the assignments to of participants to the two Sequences were balanced across demographic categories and baseline psychological and cognitive assessments, except that participants assigned to sequence RtF were slightly older than those assigned to sequence FtR (24.7 + 5.2 yr. vs 21.0 + 2.9 yr.; *p* = 0.01). Additionally, average values for the psychological and cognitive measures at intake were comparable to those observed in previously published studies of similarly aged subjects and were balanced across Sequence (Table 2) [61,62,63,64,65,67,69]. 

**Figure 4:**
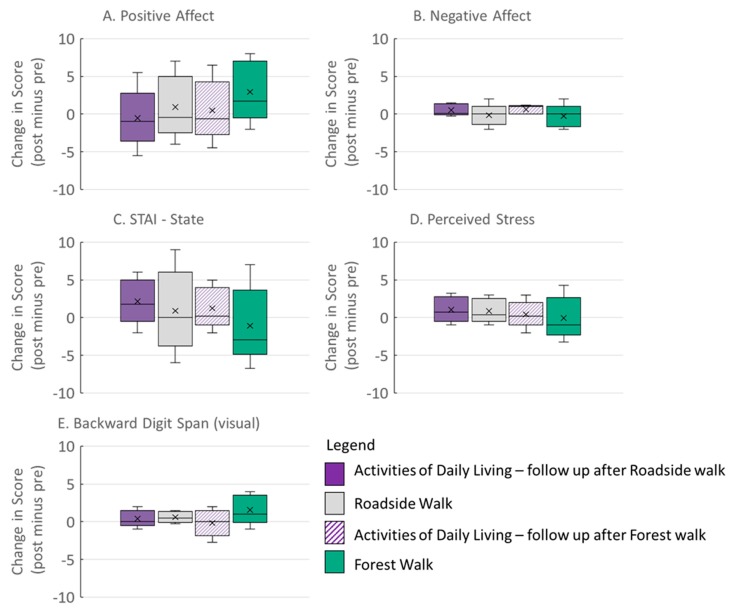
Summary of data for change in participants’ scores (post-walk score minus pre-walk score = change in score) for mood ((**A**) Positive Affect and (**B**) Negative Affect), (**C**) State anxiety, (**D**) Perceived Stress, and (**E**) Directed Attention (Backward Digit Span, visual). Box and whisker plots show the middle 50% of scores for a condition (box); the horizontal line through the middle of the box is the median, X is the mean, and the vertical lines (“whiskers”) indicate the interquartile ranges (lower 25% to upper 75%). Legends indicate the intervention experienced on the day of testing: solid purple—Activities of Daily Living one week after Roadside walk (ADL-R); gray—Roadside walk; purple lines—Activities of Daily Living one week after the Forest walk (ADL-F); solid green—Forest walk.

Mixed models linear regression was used to assess the impact of interventions on the outcomes of interest in this crossover repeated measures design study [76,77,78]. The presentation of results was guided by the guidelines for statistical reporting of the New England Journal of Medicine and recent commentaries calling for more thoughtful analysis of effect size and confidence intervals rather than reliance on probability values [79,80]. The data are summarized as medians, means, and interquartile ranges of the change in test score (post-walk score minus pre-walk score = change) in Figure 4. We used a repeated measures linear mixed-model in NCSS 8 [81] to determine whether changes in scores were dependent upon the Intervention and to estimate relative effect sizes [76,77,82]. We designated the Intervention and Series as independent variables. Intervention (forest vs. roadside walks, ADL-R and ADL-F), Series (intervention order), and Time (experiment day) were positioned as categorical fixed factors, and Participants were random factors. Changes in mood, state anxiety, perceived stress, and working memory from pre- to post-walking or pre- to post-ADL session were included as dependent variables. However, we noted no significant main effect of Series or the interaction between Intervention and Series on changes in the dependent variables (Model 1. Supplemental Table S1). Thus, Series was dropped in further analyses (Model 2. Table 3 and in Supplementary Material, Tables S2–S6).

## 3. Results

### 3.1. Psychological and Working Memory Outcomes

Forest walks had a positive impact on psychological measures. The changes in score for all outcomes are presented as Box and Whisker plots in Figure 4. Positive values for changes in Positive Affect and vBDS scores indicate improvement in mood or working memory, whereas negative values indicate improved status in all other measures. Visual inspection of Figure 3 suggests that forest walks lead to greater improvements in outcome measures than roadside walks or ADLs. Under ADL, outcome measures were either unchanged or indicated a decay in status over time. Linear mixed-models regression supported this interpretation, revealing the effects of intervention on changes in Positive Affect, Negative Affect, STAI-state, and Perceived Stress, but not working memory (vBDS) (Table 3).

Closer inspection of the mean differences among interventions (details in Supplementary Materials, Tables S2–S6) revealed that although walking leads to improved psychological state regardless of location, walks in the forest lead to greater improvements than walks on the roadside for several of the measures. Specifically, positive affect showed the greatest improvement following forest walks (Mean change [95% CI] +1.7 points [−0.6, 4.0]). However, there was a slight decrease in positive affect following roadside walks (-0.4 points [−2.1, 1.2]) and a greater decline after activities of daily living (ADL-R: −1.7 points [−3.6, 0.2]. ADL-F: −1.5 points [−2.8, −0.06]). 

Negative affect improved following forest walks, as indicated by a decrease in scores (−1.4 points [−2.3, −0.4]). Roadside walks led to smaller improvements in negative affect (−0.8 points [−2.0, 0.3]). In contrast, negative affect was slightly worse after ADLs, indicated by a positive change in score (ADL-R: 0.1 points [−1.0, 1.2]. ADL-F: 0.9 points [0.2, 1.7]). Forest walks also led to greater reductions in state anxiety than did roadside walks (Forest: −3.0 points [−5.7, −0.3]; Roadside: -1.5 points [−3.7, −0.68]). However, walks in both locations yielded improvements in contrast to ADLs for which state anxiety increased over the course of the day (ADL-R: 1.9 points; [−0.69, 4.52]. ADL-F: 1.4 points; [−0.9, 3.6]). 

As with the other measures, perceived stress was reduced following forest walks as shown by a decrease in score from pre- to post-walk (−1.4 points; [−2.6, −0.2]). There was a slight increase in perceived stress following walks along the road (0.4 points; [−0.6, 1.3]). Perceived stress showed an increase following ADL-R (0.8 points, −0.2, 1.9), but a decrease under ADL-F (−0.4 points; [−1.2, 0.4]); when combined ADL conditions reflected an increase in perceived stress (0.21 point; [−0.45, 0.87]).

Working memory, as assessed by the vBDS test, was the only measure for which linear regression did not indicate a relationship between intervention type and change in score (*p* = 0.57, Table 3). This is reflected in the similarity of the average values and the fact that the confidence intervals of the data from the four interventions all show considerable overlap with zero and with each other (Forest: 0.7 points; [−0.3, 1.6]. Roadside −0.2 points; [−0.2, 1.2]. ADL-R: −0.0 points; [−0.6, 0.6]. ADL-F: 0.3 points; [−0.5, 1.1]). 

### 3.2. Recruitment and Retention: 

Eighty people responded to the recruitment materials and took the health screening survey. Only one person was ineligible to participate based on the screening survey. Detailed information about the study protocol, time commitment, and scheduling information was sent to the 79 people who were deemed eligible for the study. After receiving this additional information, 41 (52%) of those eligible enrolled in the study. Of the first 20 people to enroll in the study 14 (70 %) were women. To increase the participation of men, new recruitment materials that specifically referred to men were prepared. Also, due to the receipt of additional funding, it was possible to increase the compensation from $U.S. 6.00/hour to $U.S. 9.00/hour. Recruitment continued until 41 participants enrolled, with enrollment of male participants prioritized during the second half of recruitment in order to obtain a final cohort with approximately equal numbers of males and females. The majority of the participants enrolled completed the entire study (N = 38, 93%). Reasons given for dropping out were unrelated to the study (one person became ill and two others developed scheduling conflicts).

## 4. Discussion

Our observations demonstrated that moderate-intensity walking in a forested environment had a positive impact on psychological health. These findings support the long-known effects of moderate-intensity physical activity on mental health and suggest that completing physical activity in greenspaces amplifies beneficial acute psychological responses. Indeed, these results echo a rich literature extolling the mental and physical health benefits of physical activity, which consistently finds that, whenever and wherever possible, 30–50 min of light exercise can buffer against the wear-and-tear of daily life [42,83]. The results also support the growing literature that activity in greenspace yields greater improvements in mental health than does activity completed indoors or in a built urban environment [43,44,52]. 

Data collected during ADL sessions was crucial for interpreting the results. Specifically, by measuring acute changes to psychological and working memory outcomes after a roadside walk, and a forest walk, and after their respective ADL sessions, we were able to parse out how moderate-intensity exercise and especially green exercise each make a positive contribution to mental health. Results from the ADL-F and ADL-R sessions showed that participants’ psychological state declined over the course of the day, possibly reflecting fatigue and stress experienced in daily life (Figure 3; Supplemental Material Tables S2–S5). When compared to ADL sessions, a walk on the forest path led to improved scores on all measures of psychological health, whereas roadside walks lead only to improvements in negative affect and state anxiety (Figure 3; Supplemental Material Tables S2–S5).

Our observation that women tended to volunteer at a higher rate than men is consistent with other studies [84,85,86,87,88,89]. We found that increasing compensation to an amount similar to the local minimum wage ($U.S. 9.00) at the time of the study and modifying recruitment materials to say that men were sought for the study increased the participation of men. However, which of these factors was responsible for the increased participation of men could not be determined. 

The protocol for Walking Green imposes a significant participant burden as compared to prior studies in three ways [46,47,48]. First multiple psychological and physiological outcomes were assessed before and after each walk. Second, the entire study lasted 29 days and required nine visits to the laboratory. Finally, participants spent as much as 3.5 hours per day engaged in the study. The fact that only 52% of those interested in the study continued to enroll after being informed of its full scope and schedule attests that these three requirements imposed a significant burden on participants.

Despite the participant burden, it is striking that the results of the current study are consistent with those reported by Berman et al. [46] in showing an improvement in mood following walks in the forest. We expand on the results of the previous study by documenting a decrease in negative affect in addition to an increase in positive affect [46]. We also expand on those findings by showing that forest walks also led to reduced state-anxiety and perceived stress, which is consistent with other studies [59,90,91]. 

However, our observations differ somewhat from those of Berman et al. [46] in that we did not see a significant improvement in working memory capacity as a result of walking location, nor did we see an improvement in working memory following walking in either location as compared to ADL. Other studies have also failed to see improvements in working memory in response to green exercise [59,92,93,94]. Three differences in the protocols may account for this difference in results between the current study and that to Berman et al. [46]. First, participants in the previous study were given a directed forgetting task to mentally fatigue them before walking, which may have magnified the difference in BDS scores before and after the walks [46]. In addition, the current protocol used the visual BDS test, whereas the previous study used the verbal BDS; the use of different modalities for the test may have led to different results [67,68,69,95]. Although several studies have assessed the impact of different modalities for administering the BDS on absolute scores, to our knowledge no study has compared the effect of test modality (visual versus verbal) on differences in scores before and after an intervention [67,68,69,95].

The third difference between the two studies is that participants in our study were driven to and from the laboratory and the start site for the walks, which required a minimum of 20 min each way. This constraint on our study protocol was intentional based on two criteria. First, we specifically selected a forested environment that was comparable to the Ann Arbor Arboretum, used in Berman et al. [46], but that location was 10 km away from the laboratory. Second, the drive time is reflective of real-world conditions and thereby makes results more generalizable. Although the driving time introduced an additional exposure between surveys and walking, it was consistent for all participants. In fact, participants who were to walk the forest and roadside routes intentionally were driven to the start site together. Finally, the heavy participant burden may have eliminated any benefit of walking or exposure to greenspace to working memory.

The fact that both the forest and roadside walks started from the same location, the parking lot of the Forest Preserve, and the fact that the roadside walk took place on a suburban roadside, may also explain why the differences between the psychological outcomes for roadside and forest walk were attenuated as compared to other studies [46,47,48,59]. Post-hoc calculation of the effect sizes for comparisons between the forest and roadside walks were small (i.e., partial η2 less than 0.03) whereas the effect sizes for comparisons among walks and ADL conditions were of medium size (i.e., partial η2 greater than 0.06) [73,96]. The studies of Berman et al., [46] Bratman et al., [47] and Park et al. [48] compared forested to urban areas with limited or no exposure to greenspace, which may have provided a greater contrast of environments than our locations. In the present study, participants experienced the trees and other greenspace associated with the Forest Preserve when arriving from and departing for the laboratory, regardless of which route they were scheduled to walk each day (Figure 3A). Second, although the Roadside route exposed participants to a heavily trafficked four lane suburban street as well as a national interstate highway, the route also contained trees and green lawns associated with business office parks (Figure 3B–D). Thus, when comparing locations with less contrasting conditions—that is, forested versus suburban rather than forested versus urban—larger sample sizes may be required.

It should be remembered that the goal is for this pilot study to be a proof of concept and refine the protocols for the Walking Green Protocol, as we initiate longitudinal studies of the health outcomes of physical activity in forested as compared to urban environments. Future studies with a larger sample size and longer term (e.g., weeks to month) longitudinal interventions will allow for more definitive conclusions about the optimal dose of nature needed to improve psychological health and cognitive functions [19,20,57,58]. A longer randomized longitudinal study could also determine the effects of nature on more chronic psychological indicators such depression or trait anxiety.

One final limitation that should be noted is that the wording for one of the response scale for the STAI was inadvertently changed from “Almost Never, Sometimes, Often, and Almost Always” to “Not at all, Somewhat, Moderately So, and Frequently So” [59,63,64]. This change in wording was not intentional and was not discovered until the study had been completed. Importantly, our participants’ baseline scores on this scale (Table 2) do not differ from normative data; thus, we have included these data in this report as indicative of the intended measure.

## 5. Conclusions

Three conclusions arise from our results: (1) a 50 min walk makes a positive impact on acute measures of mental health; (2) a 50 min walk in a forest amplifies the acute mental health benefits conferred via walking, even when participants experience a 20 min drive to and from the walk location, and (3) participant burden and gender bias in volunteerism need to be taken into account when designing recruitment strategies for longitudinal studies. Based on these results and the existing literature, we organized policy implications for each of these findings accordingly:

1. Efforts to design communities to encourage walking and active living should be supported [9,97].
2. Plans to provide access to greenspace should include public transportation to enable access to greenspace [60].
3. Healthcare professionals should counsel patients to be active, and prioritize activity in greenspaces through the use of Nature Rx [9,12,90].

Considerable research has focused on the questions of how distance and access to greenspace influence the health outcomes of urban residents, often emphasizing the importance of living near greenspace [2,11,98,99,100]. Based on this emerging literature, more parks, gardens, and natural areas should be included in city planning efforts. However, when it is not possible to place such amenities near people, our results suggest that providing opportunities for people to travel short distances to greenspace on a regular basis can be beneficial.

Walking or spending time in greenspace can be cost effective way to improve mental wellness, thus in addition to reducing stress, anxiety, and improving mood for those experiencing the wear and tear of everyday life, physical activity in greenspace may also provide an important adjunct therapy to clinical therapies [12,101,102,103,104,105]. These results support the development of evidence-based recommendations for Nature Rx to encourage people to be physically active in nature [106,107,108,109]. We believe our results and those of others provide strong support to the inclusion of greenspace in public health and urban planning [8,10,11,12]. 

## Figures and Tables

**Table 1:** Selected demographic characteristics of study participants by sequence walks (n = 38).

Characteristic	Sequence of Walks		Comparison
	Road to Forest (RtF) (n = 20)	Forest to Road (FtR) (n = 18)	Significance Level (*p*)
Sex, male (n (%))	10 (50%)	8 (44%)	0.73
Age in years (Mean (SD))	24.6 (5.3)	21.2 (2.9)	0.02
Education level (n = 37) ^1^			
Some college	11	12	0.46
College graduate	5	4	0.84
Graduate school	4	1	0.19
Employment status (n = 40) ^2^			
Full-time student	12	14	0.24
Part-time employee	3	7	0.1
Full-time employee	4	0	0.05
Ethnicity (n = 35)^1^			
African American	1	1	0.97
Asian	12	8	0.079
Hispanic	2	2	1
White non-Hispanic	5	7	1
Household income (n = 31) ^1^			
<$50,000	10	6	0.3
$50,000–$99,999	4	6	0.35
>$100,000	4	3	0.79
Childhood home location (n = 31) ^1^			
Rural	3	0	0.09
Suburban	9	11	0.32
Urban	8	6	0.67
Current home location (n = 31) ^1^			
Suburban	10	13	0.16
Urban	10	4	0.08

^1^ Sample numbers may not add up to 38 due to a lack of responses to the question. ^2^ Sample number exceeds 38 due to the selection of multiple categories.

**Table 2:** Baseline (Day 1) scores on psychological and cognitive function tests.

		Roadside First (Sequence RtF)	Forest First (Sequence FtR)	Significance Level (*p*) ^1^
Outcome Measures	Reference Values Ages 18–35 yrs	Mean (95%CI)	Mean (95%CI)	
Positive affect (10–50) ^2^	25.3–29 ^3^	29.8 (25.8, 33.9)	32.1 (29.0, 35.3)	0.36
Negative affect (10–50) ^2^	15.2–15.8 ^3^	14.8 (11.8, 17.8)	14.6 (11.2, 18.0)	0.93
State anxiety (20–80) ^2^	37.62 ^3^	36.9 (31.4, 42.4)	35.0 (29.2, 40.8))	0.62
Perceived stress (0–40) ^2^	14.2 ^3^	15.1 (11.3, 18.8)	13.7 (10.8, 16.6)	0.56
Backward digit span (visual) (0–14) ^2^	6.5–7.8 ^3^	7.3 (6.0, 8.7)	6.9 (5.6, 8.3)	0.68

^1^ Significance value calculated for the Student’s t-test comparing scores at intake (Day 1) for participants assigned to Sequences A and B; ^2^ Range of scores for the indicated scale; ^3^ Comparison values for the scales used: Positive and Negative Affect Schedule (PANAS) (momentary) for 2213 undergraduates at Southern Methodist University and 279 undergraduate students from Australian universities [61,62]; The State and Trait Anxiety Inventory (STAI)-State Average for male and female college students [63,64]; Cohen’s Perceived Stress Scale (PSS-10) average for male and female between the ages of 18 and 29 yrs [65]; Visual Backward Digit Span—range of average number of correct responses for young adults (aged 18–30 yrs) when the test is administered by computer [67,69].

**Table 3:** Results of linear mixed-models regression Model 2.

Dependent Variable (Change = Post Score − Pre Score)	Intervention Effect		
	F	DF	*p*
Positive Affect	2.5	3, 70.3	0.07
Negative Affect	3.5	6, 69.5	0.02
STAI-State	3.1	3, 57.2	0.04
PSS	2.9	3, 72.5	0.04
Backward Digit Span (visual)	0.67	3, 74.1	0.57

## Supplementary Materials

The following are available online at https://www.mdpi.com/1660-4601/16/22/4338/s1, Table S1: Results of linear mixed-models regression analysis Model 1. Table S2: Summary of results for Positive Affect. Table S3: Summary of results for Negative Affect. Table S4: Summary of results for State Anxiety. Table S5: Summary of results for Perceived Stress. Table S6: Summary of results for Visual Backward Digit Span.
