# Supplementary material for: Walking Green: Developing an Evidence Base for Nature Prescriptions
Source: Int J Environ Res Public Health. 2019 Nov 7;16(22):4338. doi: 10.3390/ijerph16224338 (PMC6888434; doi:10.3390/ijerph16224338)
Supplement: Supplementary file 1 [file ijerph-16-04338-s001.pdf]

Table S1. Results of linear mixed-models regression analysis for Change in Score using Model 1.

	Independent Variables								
	Intervention			Series			Intervention X Series		
Dependent Variable	F	DF	p	F	DF	p	F	DF	p
Positive Affect	2.6	3, 67	0.06	0.25	1, 67.1	0.62	1.4	3, 7.7	0.32
Negative Affect	**3.5**	**3, 69.2**	**0.02**	0.23	1,69.2	0.63	0.78	3, 75.6	0.51
STAIS	**3.2**	**3, 58**	**0.03**	0.08	1,58.1	0.78	1.71	3, 60.7	0.17
PSS	**3.02**	**3, 70.0**	**0.04**	0.01	1, 70.0	0.92	1.27	3, 23.8	0.31
Backward Digit Span (visual)	0.65	3, 63.2	0.59	2.12	1,63.3	0.15	0.16	3, 58.3	0.92

A preliminary analysis tested the hypothesis that Intervention, Series (the order in which the walks were taken) and the interaction between Intervention and Series influenced outcomes. There were no significant main effects of Series or the interaction between Intervention and Series; therefore, Series was dropped from the analysis (Model 2) presented in the body of the paper.

Table S2. Summary of results for Positive Affect. A. Descriptive statistics for raw scores (pre and post) and Change in Score ($Post_{i...n}$ minus $Pre_{i...n}$); increase in scores indicates improved mood. B. Results of mixed models linear regression (model 2). C. Comparison of Mean Differences of Change in Scores from linear regression model 2.

A. Intervention	n	Pre- Mean (95% CI)	Post- Mean (95% CI)	Change in Score Mean (95% CI)
ADL-R	35	27.9 (24.3, 31.4)	26.2 (22.7, 29.73)	-1.7 (-3.6, 0.2)
ADL-F	35	28.6 (25.1, 32.0)	27.4 (23.8, 30.5)	-1.5 (-2.8, -0.06)
Roadside	37	28.6 (25.4, 31.8)	28.1 (25.0, 31.1)	-0.4 (-2.1, 1.2)
Forest	36	28.3 (25.0, 31.6)	30.0 (26.3, 33.8)	1.7 (-0.6, 4.0)

B. Results for effect of intervention on Change in Positive Affect Score (Model 2). F =2.5, df 3, 70.3, p = 0.07.

C. Comparisons of Mean Differences (Effect Sizes; p value) of Change in Score from linear regression model 2.

	ADL-R	ADL-F	Roadside
Forest	3.4; 0.02	3.2; 0.02	2.0; 0.15
Roadside	1.4; 0.32	1.1; 0.42	
ADL-F	0.2; 0.86		

Table S3. Summary of results for Negative Affect. A. Descriptive statistics for raw scores (pre and post) and Change in Score ($Post_{i...n}$ minus $Pre_{i...n}$); decreased score indicates improved mood. B. Results of mixed models linear regression (model 2). C. Comparison of Mean Differences of Change in Scores from linear regression model 2.

A. Intervention	n	Pre- Mean (95% CI)	Post- Mean (95% CI)	Change in Score Mean (95% CI)
ADL-R	35	13.3 (11.7, 15.1)	13.4 (11.7, 15.2)	0.1 (-1.0. 1.2)
ADL-F	35	12.6 (11.0, 14.3)	13.6 (11.9, 15.3)	0.9 (0.2, 1.7)
Roadside	37	14.2 (12.4, 16.1)	13.0 (11.4, 14.6)	-0.8 (-2.0, 0.3)
Forest	36	13.4 (11.8, 14.9)	12.0 (11.0, 13.0)	-1.4 (-2.3, -0.4)

B. Results for effect of intervention on Change in Negative Affect Score (Model 2). F = 3.5, df = 3, 69.5, p = 0.02

C. Comparisons of Mean Differences (Effect Sizes; p value) of Change in Score from linear regression model 2.

	ADL-R	ADL-F	Roadside
Forest	-1.5; 06	-2.3; 0.004	0.6; 0.44
Roadside	-0.9; 0.24	-1.7; 0.03	
ADL-F	0.8; 0.30		

Table S4. Summary of results for State Anxiety. A. Descriptive statistics for raw scores (pre and post) and Change in Score ($Post_{i...n}$ minus $Pre_{i...n}$); decreased score indicates improved state. B. Comparison of Mean Differences of Change in Scores from linear regression model 2.

A. Intervention	n	Pre-Mean (95% CI)	Post-Mean (95% CI)	Change in Score Mean (95% CI)
ADL-R	35	34.9 (31.1, 38.7)	36.8 (32.9, 40.7)	1.9 (-0.69, 4.52)
ADL-F	35	33.4 (30.5, 36.3)	34.8 (31.8, 37.8)	1.4 (-0.9, 3.6)
Roadside	37	36.1 (32.5, 39.7)	34.4 (31.8, 36.9	-1.5 (-3.7, 0.68)
Forest	36	34.6 (30.6, 38.6)	31.6 (28.5, 34.8)	-3.0 (-5.7, -0.3)

B. Results for effect of intervention on Change in State Anxiety Score (Model 2). F = 3.1, df = 3, 57.2, p = 0.04

C. Comparisons of Mean Differences (Effect Sizes; p values) of Change in Score from linear regression model 2.

	ADL-R	ADL-F	Roadside
Forest	-4.9; 0.01	-4.3; 0.03	-1.4; 0.46
Roadside	-3.5; 0.07	-2.9; 0.13	
ADL-F	-0.6; 0.75		

Table S5. Summary of results for Perceived Stress. A. Descriptive statistics for raw scores (pre and post) and Change in Score ($Post_{i...n}$ minus $Pre_{i...n}$); decrease in score indicates improved state. B. Comparison of Mean Differences of Change in Scores from linear regression model 2.

A. Intervention	n	Pre-Mean (95% CI)	Post-Mean (95% CI)	Change in Score Mean (95% CI)
ADL-R	35	13.3 (10.9, 15.8)	14.2 (11.6, 16.8)	0.8 (-0.2, 1.9)
ADL-F	35	14.0 (11.6, 16.4)	13.7 (11.0, 16.2)	-0.4 (-1.2, 0.4)
Roadside	37	13.6 (11.2, 16.0)	13.8 (11.4, 16.2)	0.4 (-0.6, 1.3)
Forest	36	14.4 (12.0, 16.9)	13.0 (10.6, 15.5)	-1.4 (-2.6, -0.2)

B. Model 2 Results for effect of intervention on Change in Perceived Stress Scale Score. F = 2.9, df = 3, 72.5, p = 0.04

C. Comparisons of Mean Differences (Effect Sizes; p values) of Change in Score from linear regression model 2.

	ADL-R	ADL-F	Roadside
Forest	-2.2; 007	-1.0; 0.22	-1.7; 0.04
Roadside	-0.6; 0.48	0.7; 0.41	
ADL-F	-1.2; 0.13		

Table S6. Summary of results for Visual Backward Digit Span. A. Descriptive statistics for raw scores (pre and post) and Change in Score ($Post_{i…n}$ minus $Pre_{i…n}$); increase in score indicates improved state. B. Results of mixed models linear regression (Model 2). C. Comparison of Mean Differences of Change in Scores from linear regression model 2.

A. Intervention	n	Pre-Mean (95% CI)	Post-Mean (95% CI)	Change in Score Mean (95% CI)
ADL-R	33	9.3 (8.4, 10.2)	9.5 (8.6, 10.3)	-0.0 (-0.6, 0.6)
ADL-F	32	9 (8.0, 10.0)	9.3 (8.3, 10.2)	0.3 (-0.5, 1.1)
Roadside	36	8.4 (7.4, 9.4)	8.6 (7.6, 9.6)	0.5 (-0.2, 1.2)
Forest	34	8.4 (7.5, 9.3)	9.0 (8.0, 10)	0.7 (-0.3, 1.6)

B. Model 2 Results for effect of intervention on Change in Visual Backward Digit Span Score. F = 0.67, df = 3, 74.1, p = 0.57

C. Comparisons of Mean Differences (Effect Sizes) of Change in Score from linear regression model 2.

	ADL-R	ADL-F	Roadside
Forest	0.6; 0.30	0.4; 0.55	0.2; 0.79
Roadside	0.5; 0.42	0.2; 0.74	
ADL-F	-0.2; 0.65		
